# Supplementary material for: Cell swelling enhances ligand-driven β-adrenergic signaling
Source: Nat Commun. 2024 Sep 7;15:7822. doi: 10.1038/s41467-024-52191-y (PMC11379887; doi:10.1038/s41467-024-52191-y)
Supplement: Supplementary file 3 — Description of Additional Supplementary Files [file 41467_2024_52191_MOESM3_ESM.pdf]

Supplementary movie 1
cAMP measurements in adult mouse ventricular cardiomyocytes
Representative movie (out of 3 independent experiments) of ventricular cardiomyocytes isolated from CAG-Epac1-camps mice imaged on an epifluorescence microscope with a CFP (channel 1) and a YFP (channel 2) emission filters. The movie shows baseline CFP/YFP fluorescence of the Epac1-camps sensor before and after medium exchange from isotonic (Baseline) to hypotonic (Swelling).

Supplementary movie 2
Nanobody 37 recruitment to the basolateral membrane of β2-AR transfected HEK293 cells upon stimulation with isoproterenol
Representative TIRF-microscopy movie (out of 3 independent experiments) of HEK293AD cells transfected with Nb37-eYFP (channel 1) and Snap-β2-AR (channel 2) upon consecutive stimulation with 3 nM and 10 µM isoproterenol, showing a recruitment of the nanobody to the basolateral cell membrane upon agonist addition.
