# Supplementary material for: Cell swelling enhances ligand-driven β-adrenergic signaling
Source: Nat Commun. 2024 Sep 7;15:7822. doi: 10.1038/s41467-024-52191-y (PMC11379887; doi:10.1038/s41467-024-52191-y)
Supplement: Supplementary file 6 — Reporting Summary [file 41467_2024_52191_MOESM6_ESM.pdf]

Corresponding author(s): Paolo Annibale
Last updated by author(s): Aug 28, 2024

Reporting Summary

Nature Portfolio wishes to improve the reproducibility of the work that we publish. This form provides structure for consistency and transparency in reporting. For further information on Nature Portfolio policies, see our Editorial Policies and the Editorial Policy Checklist.

Statistics

For all statistical analyses, confirm that the following items are present in the figure legend, table legend, main text, or Methods section.

n/a	Confirmed	
☐	☒	The exact sample size (*n*) for each experimental group/condition, given as a discrete number and unit of measurement
☐	☒	A statement on whether measurements were taken from distinct samples or whether the same sample was measured repeatedly
☐	☒	The statistical test(s) used AND whether they are one- or two-sided<br>*Only common tests should be described solely by name; describe more complex techniques in the Methods section.*
☒	☐	A description of all covariates tested
☒	☐	A description of any assumptions or corrections, such as tests of normality and adjustment for multiple comparisons
☐	☒	A full description of the statistical parameters including central tendency (e.g. means) or other basic estimates (e.g. regression coefficient) AND variation (e.g. standard deviation) or associated estimates of uncertainty (e.g. confidence intervals)
☐	☒	For null hypothesis testing, the test statistic (e.g. *F*, *t*, *r*) with confidence intervals, effect sizes, degrees of freedom and *P* value noted<br>*Give P values as exact values whenever suitable.*
☒	☐	For Bayesian analysis, information on the choice of priors and Markov chain Monte Carlo settings
☒	☐	For hierarchical and complex designs, identification of the appropriate level for tests and full reporting of outcomes
☒	☐	Estimates of effect sizes (e.g. Cohen's *d*, Pearson's *r*), indicating how they were calculated

Our web collection on statistics for biologists contains articles on many of the points above.

Software and code

Policy information about availability of computer code

Data collection	Nikon NIS Elements, Leica LASX, Biotek Plate reader, BioTek Gen5
Data analysis	ImageJ, Prism 9, Excel 365,Visiview, MATLAB, uTrack, IgorPro 9

For manuscripts utilizing custom algorithms or software that are central to the research but not yet described in published literature, software must be made available to editors and reviewers. We strongly encourage code deposition in a community repository (e.g. GitHub). See the Nature Portfolio guidelines for submitting code & software for further information.

Data

Policy information about availability of data

All manuscripts must include a data availability statement. This statement should provide the following information, where applicable:

- Accession codes, unique identifiers, or web links for publicly available datasets
- A description of any restrictions on data availability
- For clinical datasets or third party data, please ensure that the statement adheres to our policy

Any source data will be made available by the authors upon requests.

Research involving human participants, their data, or biological material

Policy information about studies with human participants or human data. See also policy information about sex, gender (identity/presentation), and sexual orientation and race, ethnicity and racism.

Reporting on sex and gender	*Use the terms sex (biological attribute) and gender (shaped by social and cultural circumstances) carefully in order to avoid confusing both terms. Indicate if findings apply to only one sex or gender; describe whether sex and gender were considered in study design; whether sex and/or gender was determined based on self-reporting or assigned and methods used. Provide in the source data disaggregated sex and gender data, where this information has been collected, and if consent has been obtained for sharing of individual-level data; provide overall numbers in this Reporting Summary. Please state if this information has not been collected. Report sex- and gender-based analyses where performed, justify reasons for lack of sex- and gender-based analysis.*
Reporting on race, ethnicity, or other socially relevant groupings	*Please specify the socially constructed or socially relevant categorization variable(s) used in your manuscript and explain why they were used. Please note that such variables should not be used as proxies for other socially constructed/relevant variables (for example, race or ethnicity should not be used as a proxy for socioeconomic status). Provide clear definitions of the relevant terms used, how they were provided (by the participants/respondents, the researchers, or third parties), and the method(s) used to classify people into the different categories (e.g. self-report, census or administrative data, social media data, etc.) Please provide details about how you controlled for confounding variables in your analyses.*
Population characteristics	*Describe the covariate-relevant population characteristics of the human research participants (e.g. age, genotypic information, past and current diagnosis and treatment categories). If you filled out the behavioural & social sciences study design questions and have nothing to add here, write "See above."*
Recruitment	*Describe how participants were recruited. Outline any potential self-selection bias or other biases that may be present and how these are likely to impact results.*
Ethics oversight	*Identify the organization(s) that approved the study protocol.*

Note that full information on the approval of the study protocol must also be provided in the manuscript.

Field-specific reporting

Please select the one below that is the best fit for your research. If you are not sure, read the appropriate sections before making your selection.

☒ Life sciences ☐ Behavioural & social sciences ☐ Ecological, evolutionary & environmental sciences

For a reference copy of the document with all sections, see nature.com/documents/nr-reporting-summary-flat.pdf

Life sciences study design

All studies must disclose on these points even when the disclosure is negative.

Sample size	Most measurements arise from single cell imaging experiments. In this case, a sample size of at least three cells from an independent transfection, and at least three independent transfections was deemed the minimum requirement as to provide the sample size. One exception: Fig. 2 b, with 6 cells from 3 experiments for swelling and 1 single cell for control. All experiments performed in the plate reader where conducted in at least three independent transfections (plates), except for Supplementary Figs. 4f, 8, 11 and 12 , when only 2 independent experiments were performed.
Data exclusions	no data were excluded
Replication	Upon at least three independent replicates of the experiment, the results were found consistent. We shall note that our protocol refer to data acquisition and analysis that spanned over four years. Our manuscript reflects the observed reproducibility of the findings over this period
Randomization	not relevant fAs we were comparing responses under two specified conditions (swelling, not swelling). Cells (in the form of wells/coverslips) were randomly assigned to either of the two groups.or our study
Blinding	not relevant for our study

Reporting for specific materials, systems and methods

We require information from authors about some types of materials, experimental systems and methods used in many studies. Here, indicate whether each material, system or method listed is relevant to your study. If you are not sure if a list item applies to your research, read the appropriate section before selecting a response.

Materials & experimental systems

n/a	Involved in the study
☒	☐ Antibodies
☐	☒ Eukaryotic cell lines
☒	☐ Palaeontology and archaeology
☐	☒ Animals and other organisms
☒	☐ Clinical data
☒	☐ Dual use research of concern
☒	☐ Plants

Methods

n/a	Involved in the study
☒	☐ ChIP-seq
☒	☐ Flow cytometry
☒	☐ MRI-based neuroimaging

Eukaryotic cell lines

Policy information about cell lines and Sex and Gender in Research

Cell line source(s)	HEK293 were HEK-tsA201 cells (Sigma-Aldrich ECACC Cat#96121229). HEK293AD cells were from Biocat, cat. no. AD-100-GVO-CB. Gαs-KO cell line were derived from HEK293A from Thermo Fisher Scientific. HEK293 derived cells (Epac-SH187) were derived from HEK-tsA201.. hiPSCs were purchased from Coriell Institute (GM25256).
Authentication	the cell lines were not authenticated further to the purchase.
Mycoplasma contamination	Cells tested negative in the routine mycoplasma controls
Commonly misidentified lines (See ICLAC register)	No commonly misidentified cell lines were used in the study

Animals and other research organisms

Policy information about studies involving animals; ARRIVE guidelines recommended for reporting animal research, and Sex and Gender in Research

Laboratory animals	adult ventricles of CAG-Epac1-camps mice aged 8 to 12 weeks
Wild animals	*Provide details on animals observed in or captured in the field; report species and age where possible. Describe how animals were caught and transported and what happened to captive animals after the study (if killed, explain why and describe method; if released, say where and when) OR state that the study did not involve wild animals.*
Reporting on sex	n/a
Field-collected samples	n/a
Ethics oversight	All animal experiments were carried out according to the German Animal Welfare Act considering the guidelines of the NIH and the 2010/63/EU Directive of the European Parliament on the protection of animals used for scientific purposes. Animal experiments were done with approval by the Landesamt für Gesundheit und Soziales (Berlin) under the approval number G 0165/19. The animals had free access to food and water and were kept in individually ventilated cages under a 12-:12-h light/dark regime (light from 6:30 AM to 6:30 PM), a constant 22 °C ± 2 °C temperature, and 55 ± 10% humidity.

Note that full information on the approval of the study protocol must also be provided in the manuscript.
